# Improvement in indices of cellular protection after psychological treatment for social anxiety disorder

**DOI:** 10.1038/s41398-019-0668-2

**Published:** 2019-12-19

**Authors:** Kristoffer N. T. Månsson, Daniel Lindqvist, Liu L. Yang, Cecilia Svanborg, Josef Isung, Gustav Nilsonne, Lise Bergman-Nordgren, Samir El Alaoui, Erik Hedman-Lagerlöf, Martin Kraepelien, Jens Högström, Gerhard Andersson, Carl-Johan Boraxbekk, Håkan Fischer, Catharina Lavebratt, Owen M. Wolkowitz, Tomas Furmark

**Affiliations:** 1 Centre for Psychiatry Research, Department of Clinical Neuroscience, Karolinska Institutet, Stockholm, Sweden; 2 Department of Psychology, Stockholm University, Stockholm, Sweden; 3 Department of Psychology, Uppsala University, Uppsala, Sweden; 4 Department of Clinical Sciences Lund, Psychiatry, , Lund University, Lund, Sweden; 5 Department of Molecular Medicine and Surgery, Karolinska Institutet, Stockholm, Sweden; 6 Center for Molecular Medicine, Karolinska University Hospital, Stockholm, Sweden; 7 Centre for Psychiatry Research, Department of Clinical Neuroscience, Karolinska Institutet, & Stockholm Health Care Services, Region Stockholm, Stockholm, Sweden; 8 Department of Clinical Neuroscience, Karolinska Institutet, Stockholm, Sweden; 9 Stress Research Institute, Stockholm University, Stockholm, Sweden; 10 Department of Behavioural Sciences and Learning, Linköping University, Linköping, Sweden; 11 Centre for Demographic and Ageing Research, Umeå University, Umeå, Sweden; 12 Center for Magnetic Resonance (DRCMR), Centre for Functional and Diagnostic Imaging and Research, Copenhagen University Hospital, Hvidovre, Denmark; 13 Department of Psychiatry, University of California, San Francisco, CA USA

**Keywords:** Human behaviour, Personalized medicine

## Abstract

Telomere attrition is a hallmark of cellular aging and shorter telomeres have been reported in mood and anxiety disorders. Telomere shortening is counteracted by the enzyme telomerase and cellular protection is also provided by the antioxidant enzyme glutathione peroxidase (GPx). Here, telomerase, GPx, and telomeres were investigated in 46 social anxiety disorder (SAD) patients in a within-subject design with repeated measures before and after cognitive behavioral therapy. Treatment outcome was assessed by the Liebowitz Social Anxiety Scale (self-report), administered three times before treatment to control for time and regression artifacts, and posttreatment. Venipunctures were performed twice before treatment, separated by 9 weeks, and once posttreatment. Telomerase activity and telomere length were measured in peripheral blood mononuclear cells and GPx activity in plasma. All patients contributed with complete data. Results showed that social anxiety symptom severity was significantly reduced from pretreatment to posttreatment (Cohen’s *d* = 1.46). There were no significant alterations in telomeres or cellular protection markers before treatment onset. Telomere length and telomerase activity did not change significantly after treatment, but an increase in telomerase over treatment was associated with reduced social anxiety. Also, lower pretreatment telomerase activity predicted subsequent symptom improvement. GPx activity increased significantly during treatment, and increases were significantly associated with symptom improvement. The relationships between symptom improvement and putative protective enzymes remained significant also after controlling for body mass index, sex, duration of SAD, smoking, concurrent psychotropic medication, and the proportion of lymphocytes to monocytes. Thus, indices of cellular protection may be involved in the therapeutic mechanisms of psychological treatment for anxiety.

## Introduction

Psychiatric disorders have been linked to increased risks of somatic illnesses and even premature death^1^, although the underlying mechanisms are not clear. Recent studies raise the possibility that accelerated cellular aging and deficiencies in cellular protection contribute to this association^2,3^. An often-used indicator of accelerated cellular aging is telomere length measured in blood leukocytes. Telomeres protect the chromosomes from damage and shorten with age^4^. They are restored by the major telomere-lengthening enzyme telomerase^5,6^. Oxidative stress also contributes to accelerated cellular aging and cellular apoptosis^7–9^, and this process can be counteracted by antioxidant enzymes, such as glutathione peroxidase (GPx)^10^. A large study including >1200 participants found that anxiety-disordered patients, relative to healthy controls, exhibit shorter mean leukocyte telomere length^11^. This is in alignment also with other studies on disorders of the anxiety and affective spectrum^12,13^, including depression and posttraumatic stress disorder—for a review see Darrow et al.^14^. Thus, it is likely that accelerated cellular aging is involved in the pathophysiology of anxiety. Preclinical data suggest that these effects could be modulated by a compensatory activation of telomerase or GPx^10,15^. Moreover, telomerase activation may represent a biological mechanism mediating treatment efficacy of various psychotropics, including antidepressants^15^, lithium^16^, and antipsychotics^17^. A small-scale study showed that antidepressant response to a selective serotonin reuptake inhibitor (SSRI) was associated with lower pretreatment telomerase and a greater increase in telomerase activity over the course of treatment^18^. Markers of oxidative stress have also been related to treatment outcome^19^, e.g., increased oxidative stress is associated with poorer response to antidepressants^20^. There are also studies in healthy individuals proposing that meditation training may be associated with increased mononuclear cell telomerase activity^21–23^, and telomerase-stress reduction relationships have also been reported in obese women after mindfulness training^24^. Moreover, human and animal studies suggest that physical exercise increases telomerase activity^25^. It is also possible that beneficial effects of psychosocial interventions are linked to reduced oxidative stress. For instance, there are studies showing reductions in oxidized low-density lipoprotein after cognitive behavioral therapy (CBT) for sleep disturbance^26^ and major depression^27^. There are early reports suggesting a decrease in GPx after SSRI treatment for major depression^28^ and social anxiety^29^, while more recent studies have not found SSRI-associated GPx changes after short-^30,31^ or long-term^32^ treatment of depression. Thus, previous studies on this topic are still scarce, treatments and study populations vary, and findings have been mixed.

In sum, there is a growing literature pointing to the importance of telomere biology in psychiatric disorders and that telomere-related cellular protection may be targeted by psychosocial and behavioral interventions. To our knowledge, no previous study has investigated changes in telomerase and GPx activity after psychological treatment for a psychiatric disorder.

Anxiety disorders, including social anxiety disorder (SAD), are highly prevalent and debilitating psychiatric conditions^33^. In the US, the estimated lifetime prevalence is 28.8% for anxiety disorders and 12.1% for SAD^34^. Among currently available treatment options, CBT and SSRIs are efficacious^35^. However, despite robust effects^35,36^, a large proportion, ~40–50% of patients with SAD, has been reported to be either treatment resistant or not responding sufficiently to current treatments^37^. Identifying biomarkers involved in the anxiolytic response could potentially improve current clinical practice by providing targets for novel interventions and increase treatment precision. CBT delivered via the internet is a promising strategy to treat patients with SAD, and there is evidence suggesting that internet-delivered CBT is as effective as conventional CBT for this condition^38^. Internet-delivered interventions provide an excellent research context for clinical trials, e.g., with respect to facilitated monitoring of treatment compliance and progress^39^. We have previously evaluated internet-delivered CBT for SAD by use of functional and structural magnetic resonance imaging (MRI)^40,41^, noting a CBT-related reduction of amygdala activity as previously demonstrated for traditional CBT and SSRI pharmacotherapy^42^. Thus, robust effects can be expected from internet-delivered CBT for SAD, and this treatment can be used as a means to further our understanding of the biological mechanisms of action in psychological treatments.

The purpose of the present study was to investigate the relationship between telomerase and GPx enzyme activities, relative telomere length, and social anxiety symptom reduction after psychological treatment for SAD. We hypothesized that the response to CBT would be associated with improved cellular protection, as indexed by increased telomerase and GPx enzyme activities.

## Materials and methods

The study was registered at ClinicalTrials.gov (id: NCT01312571), and approval was obtained from the regional ethics committee at Umeå University, Sweden (dnr: 2015-310-31 M). All participants gave written informed consent prior to participation.

## Participants

Media ads targeting individuals experiencing social anxiety were used for recruitment, and individuals responded by answering online questionnaires on demographics and social anxiety, including the Liebowitz Social Anxiety Scale Self-Report (LSAS-SR)^43^. All participants were at least 18 years of age, had no neurological disorder, no concurrent psychological treatment and if treated with a psychotropic medication, they agreed to maintain a stable dose at least 3 months before enrollment and during treatment in the current study. All participants also met MRI safety criteria, e.g., not being pregnant and having no ferromagnetic object in the body.

A total of 51 patients with SAD were recruited, but after dropout (*n* = 2) and incidental findings after initial MRI (*n* = 3), 46 participants entered treatment. All of them remained for the final assessments. Before treatment and baseline assessments, the patients were interviewed via telephone using the full Mini-International Neuropsychiatric Interview (M.I.N.I.) version 7.0 and the social phobia section of the Structured Clinical Interview for DSM-IV—Axis I Disorders (SCID-I). The patients had to meet the diagnostic criteria for SAD as their principal diagnosis. Patients were excluded if they had a severe ongoing depression (as indexed by scoring > 34 on the self-rated Montgomery Åsberg Depression Rating Scale, MADRS-S)^44^, current bipolar or psychotic disorders, current alcohol or substance use disorders, or antisocial personality disorder. Other acute illness was not an exclusion criterion.

Four (9%, *n* = 4/46) patients were on concurrent psychotropic medication, i.e., stable dosage of SSRIs that did not change throughout the study period. Two (4%, *n* = 2/46) patients had previously used beta blockers in social situations but agreed not to use them during the study period. Seventeen (37%, *n* = 17/46) had previous experience with some form of psychological treatment but no one had an ongoing therapy. Also, the patients did not change their level of physical exercise during the study, as assessed with a single open-ended question. At the time of recruitment, one patient was on short-term sick leave due to non-psychiatric reasons (and was later not identified as an outlier on any biomarker), all the others were currently employed or students. See Table 1 for a detailed summary of descriptive characteristics. Power calculation based on the main finding in Wolkowitz et al.^18^ (telomerase activity change correlating with change in depressive symptoms), suggest that the achieved power in this study was above 99%.

**Table 1:** Demographics, clinical status, concurrent medications, and comorbid conditions in the sample.

Variable	Social anxiety disorder patients, *n* = 46
Female gender, *n* (%)	29 (63.0)
Age (mean ± s.d.)	30.7 ± 8.3
SAD, duration in years (mean ± s.d.)	17.0 ± 9.9
BMI (mean ± s.d.)	24.8 ± 4.4
Smoking regularly the past 3 months, *n* (%)	4 (9.0)
Marital status, *n* (%)	
Married/cohabiting with children	16 (34.8)
Married/cohabiting without children	10 (21.7)
Noncohabiting partner	4 (8.7)
Single with children	4 (8.7)
Single without children	9 (19.6)
Other	3 (6.5)
Education, *n* (%)	
Completed primary school	3 (6.5)
Completed secondary school	7 (15.2)
Completed vocational education	2 (4.3)
Ongoing university education	16 (34.8)
Completed university education	16 (39.1)
Concurrent medications, *n* (%)	
No medication	18 (39.1)
SSRIs	4 (8.7)
Hormonal contraceptives	16 (34.8)
Antihistamines	1 (2.2)
Hormone medications	1 (2.2)
Thyroid hormone substitution	2 (4.4)
Concurrent psychiatric comorbidity (M.I.N.I.), *n* (%)	
No concurrent psychiatric comorbidity	12 (26.1)
Major depressive disorder	3 (6.5)
Panic disorder	3 (6.5)
Agoraphobia	5 (10.9)
Generalized anxiety disorder	3 (6.5)
Binge eating disorder	1 (2.2)
Obsessive compulsive disorder	2 (4.3)

*BMI* body mass index, *M.I.N.I.* Mini-International Neuropsychiatric Interview, *SAD* social anxiety disorder, *SSRIs* selective serotonin reuptake inhibitors

### General procedure and design

This study used a within-subject design including screening, and two baseline assessments before treatment initiation (pretreatment), and one assessment at posttreatment. Multiple baseline assessments were included to control for standard confounds related to time and measurements, like regression to the mean, repeated testing, and spontaneous remission. Two baseline assessments were separated by 9 weeks, which equals the number of weeks of the current intervention. All 46 patients contributed with complete clinical and biomarker data, and there were no missing data on the primary clinical outcome measure.

### Clinical assessment

The LSAS-SR is a 24-item self-report questionnaire used internationally to assess treatment-related changes in social anxiety symptoms^45^. LSAS-SR was the primary outcome and all patients completed assessments at screening, first and second baseline, and posttreatment.

### Internet-delivered CBT

Internet-delivered CBT for SAD has been described extensively elsewhere^46,47^. Relative to conventional CBT, patients should go through the same behavioral changes, i.e., only the administration format differs. Briefly, the internet-delivered CBT was a guided self-help intervention lasting 9 weeks^47^. Each week, the patients were provided with a module containing text and homework assignments based on CBT. The content was standardized, i.e., all were provided with the same material, and identical to our previous randomized controlled trials (RCTs), e.g., refs. ^47,48^. Exposure-based exercises, a main treatment component, were introduced midway, i.e., in the fifth module of the treatment. Patients were in weekly contact with a clinical psychologist providing written feedback and guidance via a secured platform via the Internet. The clinical psychologist provided feedback on the homework assignments, and the patients undertook a weekly test with questions related to CBT and the content of the modules. To control for adherence, the patients had to give 100% correct responses on the multiple-choice questionnaire (with the possibility of redoing the test multiple times). After completion of the homework assignments and the multiple-choice quiz, the next module was made available to the patient.

### Clinical psychologists

Seven clinical psychologists were therapists in the current study. Five (*n* = 71.4%) were licensed clinical psychologists and two (*n* = 28.6%) were clinical psychology students in their final year and received clinical supervision. At posttreatment, the therapists gave subjective reports on each patient’s compliance to the exposure exercises, and the ratings were “no/minor”, “satisfactory”, or “to a large extent”.

### Assays

Experienced research nurses collected all blood samples in the morning (from 7:00 a.m. to 11:30 a.m.) after patients had been fasting since 10:00 p.m. the night before. To ensure compliance, all the patients received a mobile phone text message the day before to remind about fasting instructions. The patients were resting for 15 min before the blood was obtained.

Whole blood was obtained in 8 ml BD Vacutainer® CPT™ Mononuclear Cell Preparation Tube—Sodium Citrate (Becton Dickinson). Plasma and mononuclear cells, being lymphocytes and monocytes, were separated within 15 min to 2 h of sampling according to manufacturer’s protocol. Briefly, whole blood was centrifuged for 20 min at 1500 *g*, whereafter plasma and the mononuclear cell layer were separated and centrifuged again for 15 min at 300 *g* with phosphate-buffered saline added to the cells for washing. The plasma was immediately frozen at –80 °C. Approximately, half of the pelleted cells were lysed by incubation with 120 µl CHAPS (Merck Millipore, including 0.15 units/µl RiboLock [LifeTechnologies, Thermo Fisher Scientific]) on wet ice for 30 min and three short vortexes, thereafter the lysate was stored at –80 °C.

Approximately, half of the pelleted cells were stored at –80 °C until DNA extraction. Genomic DNA was extracted using DNeasy® Blood & Tissue Kit (Qiagene), with a modified protocol to reduce DNA shearing. Briefly, cell lysis was done at 37 °C for 3 h, vortex was avoided, and centrifugations were performed at 6000 *g*^49^. DNA concentration was quantified with NanoDrop ND-1000 Spectrophotometer (Nano-Drop Technologies Inc., Wilmington, DE, USA).

Differential leukocyte counts were determined in whole blood collected in ethylenediaminetetraacetic acid (EDTA) tubes by the University Hospital of Umeå.

### Telomerase activity assay

Telomerase activity was assayed by modified real-time telomeric repeat amplification protocol^50^. Samples, controls, and standard curve dilutions were run in triplicate, and standard curve and controls were present on all plates. All samples from each patient were run on the same plate. Efficiency was 95–101%. The mean of the correlation coefficients of the standard curves were all above 0.98. The coefficients of variation (CV) of intra-assay Ct values for the standard dilutions of the four plates was 1.0% and inter-assay was 0.47%. The detection success rate was 100%, and all samples were run in the same batch. See also Supplementary Material.

### GPx activity assay

GPx activity was determined using BioVision Glutathione Peroxidase Activity Colorimetric Assay Kit (Catalog#K726-100) according to the protocol, where the GPx activity was calculated using an NADPH standard curve. The correlation coefficients of the standard curves were all above 0.99. The inter-plate CV of GPx activity was 6.3% and the within-plate CV was 4.4% calculated from positive control run in nine 96-well plates. The assay success rate was 100% (*n* = 46 at each timepoint), and all samples were run in the same batch. See also Supplementary Material.

### Relative telomere length assay

Relative telomere length was determined using real-time quantitative PCR according to Cawthon et al.’s protocol^51^, where the relative telomere to single copy gene (T/S) ratios was determined using a standard curve. In brief, each DNA sample (10 ng) was assessed for the telomere and the single-copy gene (hemoglobin-b, *HBB*) in triplicate within the same 384-well plate, amplified by using Platinum® SYBR® Green and 0.5 µM of each primer in 10 µl total reaction volume. The correlation coefficients of the standard curves were above 0.99 for each primer set and 384 plate. The inter-plate CV of T/S ratio was 6.3% calculated from a patient sample run in four 384-well plates. The detection success rate was 100%. Samples from all three timepoints per individual were assayed in the same 384-well plate. See also Supplementary Material.

### Possible confounders

There is evidence that smoking, sex, and body mass index (BMI) are linked to leukocyte telomere length, i.e., smokers^52^, males^53^, and high BMI^54^ are associated with shorter telomeres. Changes in telomerase activity are associated with antidepressant response^18^. Also, in a large study duration of depressive disorder was inversely associated with telomere length^55^. In the current study, we were interested in the psychological treatment response in relation to biomarkers, and thus, smoking, BMI, concurrent SSRIs, and duration of illness were considered possible confounders. For calculations of pre–post change in telomerase and telomere length showing statistical significance, change in the proportion of lymphocytes to monocytes was added as a nuisance variable^56^.

### Statistics

Demographic, clinical, and biomarker statistics were evaluated using the STATA Statistical Software, v. 15.0 (STATA Corporation, College Station, TX, USA).

Generalized Estimating Equations (GEE) with exchangeable correlation structure were used to evaluate panel data on treatment effects across time. Linear regression models were used to assess associations between biomarkers and clinical outcome. The linear regression models including biomarkers and symptom scores were checked for outliers using measures of influence (Cook’s *D*)^57^, discrepancy (studentized residuals), and Hosmer–Lemeshow leverage. Individual values with both high influence (Cook’s *D* > 4/46 = 0.09) and either high residuals (±3) or leverage (>2/46 = 0.04) were determined to be outliers. One patient’s baseline GPx, and two patients’ baseline telomerase were outliers. Three patients’ telomerase change, two patients’ GPx change, and two patients’ change in telomere length were determined to be outliers and excluded from further linear regression. To account for normality violations in biomarkers, nonparametric bootstrapping (×1000) was used to estimate standard errors (Bootstrapped Standard Errors) in the GEE’s and linear regression models.

Within-group effect sizes (Cohen’s *d*) were based on observed values and calculated by dividing the mean difference with respective standard deviations and correction for the correlation between timepoints. Pre–post change on LSAS-SR was determined by calculating each patient’s change score from pretreatment (mean of the screening, the first and second baseline assessment) to posttreatment [posttreatment – (mean baseline)]. Similarly, the change scores are reported for biomarkers. BMI, sex, duration of SAD, smoking, and concurrent SSRI-treatment were added as covariates in multiple regressions, including the respective biomarker.

## Results

### Main effect of treatment on social anxiety symptoms

#### Self-reported social anxiety

**Fig. 1:**
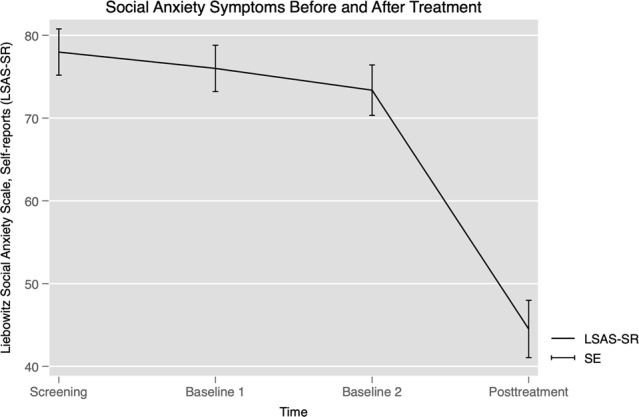
Changes in social anxiety symptom severity as assessed with the Liebowitz Social Anxiety Scale, self-rated version (LSAS-SR), before and after the treatment (screening, first and second baseline, and at post-treatment). LSAS-SR scores from screening did not change significantly to the first (*Z* = 0.78, *p* = 0.438) or second baseline (*Z* = 1.81, *p* = 0.071). As expected, LSAS-SR scores decreased markedly (*B* = –33.46, *Z* = 13.13, *p* < .001) from pretreatment to posttreatment (LSAS-SR mean = 44.52, s.e. = 3.00, 95% CI 38.64, 50.41). Error bars represent the standard error.

As shown in Fig. 1, LSAS-SR scores from screening (LSAS-SR mean = 77.98, s.e. = 3.00, 95% confidence interval (CI) 72.09, 83.86) did not change significantly to the first (*Z* = 0.78, *p* = 0.438) or second baseline (*Z* = 1.81, *p* = 0.071). As expected, LSAS-SR scores decreased markedly (*B* = –33.46, s.e. = 2.55, *Z* = 13.13, *p* < 0.001) to posttreatment (LSAS-SR mean = 44.52, s.e. = 3.00, 95% CI 38.64, 50.41). The within-group Cohen’s *d* effect size for improvement (difference between the mean of screening, first and second baseline, and posttreatment) was large (*d* = 1.46; 95% CI 1.05, 1.87).

#### Treatment compliance

The mean (±s.d.) number of completed treatment modules was 7.8 (±1.8), and 78.3% (*n* = 36/46) of the patients completed at least seven out of nine modules. Six patients did not complete the fifth module in which the exposure interventions were initiated; thus, 40 patients undertook exposure interventions. The clinical psychologists estimated that 35.0% (*n* = 14/40) completed “some” exposure exercises, while 47.5% (*n* = 19/40) performed exposure exercises “to a large extent”.

Across all patients, there was an association between compliance to exposure exercises and reduction of LSAS-SR symptoms (pretreatment vs posttreatment; Adj-*R*^2^ = 0.08, *β* = –0.31, *B* = –6.19, BSE = 2.47, *Z* = 2.51, *p* = 0.012). LSAS-SR at baseline was not associated with compliance to exposure (*β* = –0.05, *p* = 0.729).

#### Pretreatment telomerase, GPx, and relative telomere length as predictors of treatment response

Pretreatment telomerase activity was significantly associated with pre–post changes in LSAS-SR (Adj-*R*^2^ = 0.10, *β* = 0.35, *B* = 27.34, BSE = 11.35, *Z* = 2.41, *p* = 0.016), with lower levels of telomerase activity predicting greater improvement in social anxiety. This association remained after adding the proportion of lymphocytes to monocytes (*p* = 0.024), BMI (*p* = 0.021), sex (*p* = 0.031), years with SAD (*p* = 0.009), smoking (*p* = 0.018), and concurrent SSRIs (*p* = 0.013) as nuisance variables. Neither pretreatment GPx activity (*β* = 0.19, *Z* = 1.53, *p* = 0.127) nor pretreatment telomere length (*β* = 0.02, Z = 0.14, *p* = 0.890) were significantly associated with pre–post change in LSAS-SR symptoms. Furthermore, as implemented in regression models with age as a covariate of no interest, telomerase (*p* = 0.906), GPx (*p* = 0.900), and telomere length (*p* = 0.870) at baseline, were not associated with length of illness, i.e., number of years with SAD.

#### Changes in telomerase and GPx activities, and telomere length over the course of treatment

**Fig. 2:**
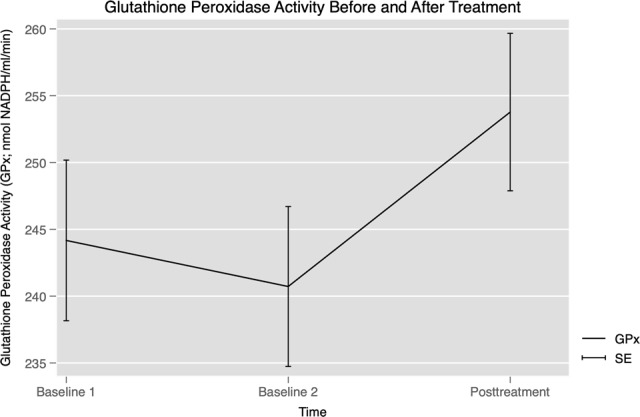
Glutathione peroxidase activity (GPx) was stable across the two baselines (*Z* = 1.25, *p* = 0.213), but increased significantly from pretreatment to posttreatment (*Z* = 2.87, *p* = 0.004). Error bars represent the standard error.

Telomerase activity was stable from first to second baseline (*B* = 0.05, BSE = 0.05, *Z* = 1.16, *p* = 0.246), and to posttreatment (*B* = 0.03, BSE = 0.07, *Z* = 0.48, *p* = 0.633). GPx activity was stable across the two baselines (*B* = –3.45, BSE = 2.77, *Z* = 1.25, *p* = 0.213), but then increased significantly to posttreatment (*B* = 9.60, BSE = 3.34, *Z* = 2.87, *p* = 0.004; see Fig. 2). After adding covariates (i.e., BMI, sex, years with disorder, smoking, and concurrent SSRIs), the pre-to-post increase in GPx remained significant (*p* < 0.006). Relative leukocyte telomere length did not change from the first to second baseline *B* = 0.01, BSE = 0.03, *Z* = 0.31, *p* = 0.753; or to posttreatment, *B* = –0.01, BSE = 0.03, *Z* = 0.32, *p* = 0.746.

#### Associations between telomerase, GPx, relative telomere length, and treatment response

##### Telomerase activity and treatment response

**Fig. 3:**
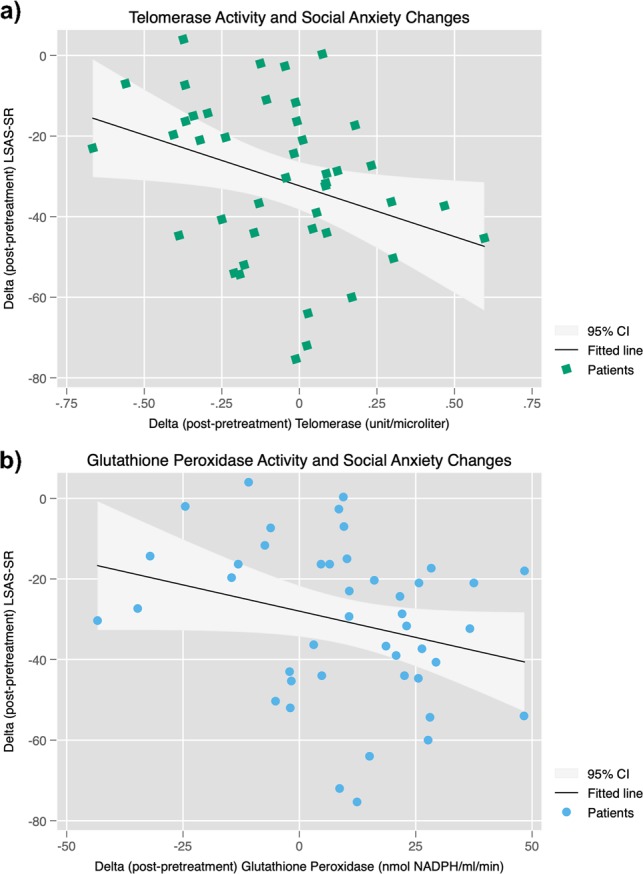
Telomerase and glutathione peroxiades changes associated with treatment response. **a** Negative association between change in telomerase activity (pre–post) and corresponding change in the Liebowitz Social Anxiety Scale, self-rated version (LSAS-SR; *β* = –0.33, *Z* = 3.22, *p* = 0.001), indicating greater increases in telomerase activity in patients with more reduced social anxiety after treatment. The telomerase activity is given as (units/µl) in 0.588 µg of total protein ([3.68 × 8] / 50). **b** Negative association between change in GPx activity (pre–post) and corresponding change in the LSAS-SR (*β* = –0.28, *Z* = 2.34, *p* = 0.024), indicating greater increases in GPx activity in patients with more reduced social anxiety after treatment.

Pre–post change in telomerase activity was negatively associated with pre–post change in LSAS-SR, indicating that patients with increases in telomerase activity over the course of treatment exhibited greater reduction of social anxiety symptoms (Adj-*R*^2^ = 0.09, *β* = –0.33, *B* = –25.21, BSE = 7.82, *Z* = 3.22, *p* = 0.001); Fig. 3a. Change in telomerase activity remained significantly associated with pre–post change in LSAS-SR also after adding baseline LSAS-SR severity to the regression model (Adj-*R*^2^ = 0.16, *Z* = 2.97, *p* = 0.003).

##### GPx activity and treatment response

Pre–post change in GPx activity was negatively associated with pre–post change in LSAS-SR, indicating that patients with increased GPx activity showed greater reduction of social anxiety (Adj-*R*^2^ = 0.05, *β* = –0.28, *B* = –0.26, BSE = 0.11, *Z* = 2.34, *p* = 0.024; Fig. 3b). Change in GPx activity remained significantly associated with pre–post change in LSAS-SR also after adding baseline LSAS-SR severity to the regression model (Adj-*R*^2^ = 0.08, *Z* = 2.19, *p* = 0.029).

##### Relative telomere length and treatment response

Pre–post change in relative telomere length was not significantly associated with pre–post change in LSAS-SR (Adj-*R*^2^ < 0.01, *β* = 0.14, *B* = 26.22, BSE = 26.11, *Z* = 1.00, *p* = 0.315).

##### Concurrent changes in telomerase, GPx enzyme activities and relative telomere length

Pre–post changes in telomerase and GpX activity were not significantly associated (*β* = 0.08, *p* = 0.565). Similarly, telomerase (*β* = –0.05, *p* = 0.781) or GpX (*β* = 0.04, *p* = 0.844) were not significantly associated with telomere length alterations from pretreatment to posttreatment. Because both telomerase and GPx changes were associated with LSAS-SR symptom change, both were included in the same multiple regression model, which also included telomere length changes. Alterations in telomerase and GPx activities were still independently and significantly associated with pre–post change in LSAS-SR (Adj-*R*^2^ = 0.26; telomerase: *β* = –0.32, *B* = –22.47, BSE = 6.64, *Z* = 3.38, *p* = 0.002; GPx: *β* = –0.34, *B* = –0.30, BSE = 0.11, *Z* = 2.78, *p* = 0.009). Importantly, after adding the covariates, i.e., BMI (*p* ≤ .010), sex (*p* ≤ .019), years with SAD (*p* ≤ .012), smoking (*p* ≤ .004), concurrent SSRIs (*p* ≤ .017), and pre–post change in proportion of lymphocytes to monocytes (*p* ≤ 0.020), the significant relationships remained between changes in LSAS-SR symptoms and telomerase and GPx activity, respectively.

## Discussion

In this longitudinal study, indices of cellular aging and oxidative stress protection were assessed twice before, and immediately after a psychological treatment (CBT) for a common psychiatric disorder (SAD). A large within-group treatment effect (Cohen’s *d* = 1.46) was observed on the primary social anxiety measure, indicating substantial symptom improvement that exceeded effects reported in our previous RCTs on internet-delivered CBT for SAD, e.g., refs. ^47,48,58^. We found that putatively enhanced cellular protection, as indexed by increases in activity of the telomere-preserving enzyme telomerase and antioxidant enzyme GPx, paralleled social anxiety reduction. Also, pretreatment telomerase activity was predictive of symptom improvement. Although enzyme activities did not increase in all patients, those who showed the greatest clinical improvement were more likely to show increases in telomerase or GPx activity over the course of treatment. Interestingly, anxiety-related changes in GPx and telomerase activity were statistically unrelated, suggesting at least partially independent cellular mechanisms underlying the beneficial effects of treatment. GPx is primarily involved in redox homeostasis as well as several other important functions^59^, whereas, telomerase is involved in telomere maintenance and a wide variety of extratelomeric functions, including suspected neuroprotective and antidepressant-like effects. The intracellular effects of GPx involve enzymatic reduction of hydrogen peroxide to water, thereby limiting its harmful effects^60^. Reactive oxygen species including hydrogen peroxide are also involved in other cellular processes, such as mitochondrial function and growth factor-mediated cell signaling, thus GPx may also affect such aspects of cellular function^60^.

Telomere maintenance, telomerase, and oxidative stress have complicated intracellular relationships. Telomeres are particularly sensitive to shortening under conditions of oxidative stress^61^. Telomerase re-extends telomeres, but under conditions of oxidative stress, it shuttles from the nucleus to the mitochondria^62–64^, where it protects mitochondria biogenesis and decreases oxidative stress, partly by potentiating cellular antioxidant defense systems, such as by increasing the ratio of reduced (GSH) to oxidized (GSSG) glutathione^65^ that is catalyzed by GPx and glutathione reductase activities. Thus, telomerase and GPx are expected to have cellular protective effects, either at the locus of the nucleus or the mitochondria. Apart from affecting redox homeostasis, GPx is also involved in several other pathways^59^, making it difficult to ascribe particular functions to its role in the present findings. Due to limitations in our ability to pinpoint the subcellular localization of these enzymes in different clinical conditions, it would be only speculative to posit specific intracellular mechanisms related to the effects we report. Increases in both telomerase and GPx activities could reflect a more favorable cellular milieu and it is possible that these functions are linked to symptom improvement following CBT. This is, to the best of our knowledge, the first study to demonstrate evidence of increased cellular protection indices in psychiatric patients after a psychosocial intervention.

The current findings are in line with Wolkowitz et al.^18^ showing that both lower baseline telomerase activity, and greater increase in telomerase activity over 8 weeks of SSRI treatment were associated with superior antidepressant response in patients with major depression. It has been speculated that improved telomerase activity is a possible mechanism of action by psychotropic medications^17^. Here, we found that a psychological treatment targeting a common anxiety disorder was associated with increases in telomerase activity, suggesting that also nonpharmacological interventions for psychiatric conditions may influence indices of cellular aging. It is possible that the relationship between increased telomerase activity and successful treatment response cuts across different treatment modalities and diagnostic groups, although it is still unknown whether this is a causal mechanism. With regards to behavioral change, physical exercise can affect cellular aging and telomerase activity in the healthy individuals without a psychiatric disorder (for a review see Deng et al.)^25^. The patients in the current study did not change their level of physical exercise throughout the study period, making this confound unlikely. Our results partly support the hypothesis that accelerated cellular aging in individuals with psychiatric conditions could be a reversible process^66^, and that some of these effects, to the extent they exist, may be mediated via mechanisms involving telomerase and/or an antioxidant enzyme systems. The short-term increases in telomerase and GPx activations were not significantly related to telomere length. Telomeres are dynamic and changes in leukocyte telomere length can be detected after a few months^67^. The current CBT was in total 9 weeks, thus, this time frame is too narrow to determine treatment-related telomere length alterations. Changes in relative telomere length in association with cellular protection and reductions in social anxiety, needs to be investigated with long-term follow-ups.

Treatment-associated increases in the antioxidative stress marker GPx activity were related to reduced social anxiety. There is previous evidence demonstrating CBT-associated oxidative stress reductions in relation to improved mental health^26,27^. Thus, it is possible that the antioxidant GPx is linked to reduced anxiety and oxidative stress. In the present study, we did not, however, measure oxidative stress substrate markers, such as F2-isoprostanes, 8-OHdG, or malondialdehyde. There are some suggestions that GPx activity is directly, rather than inversely, correlated with oxidation by-products, signifying a homeostatic response to elevated oxidative stress^68^. In the absence of other measures, it is not known if the observed increases in GPx activity with treatment are correlated with increased or decreased oxygen/nitrogen free radicals. Thus, we were not able to determine net changes in oxidative status^20,69^. There have been mixed findings regarding therapeutic responses and GPx activity alterations, with reports of no changes, increases and reductions^20,29,31^. We assayed GPx activity in plasma, whereas some other studies testing the relationship between GPx and antidepressant response assessed GPx activity in hemolyzed erythrocytes^28,29^. Although the significance of differences between these two compartments are not fully understood, they are genetically and immunologically distinct and show different physical and kinetic properties^70^. Thus, our GPx results cannot uncritically be compared with the results of some previous studies.

This study included multiple and complete biological assessments of all treated patients and we had no patients dropping out from CBT. However, among the limitations it should be noted that our study was not an RCT, thereby lacking active treatment or psychological placebo control groups. This is partly because the treatment program has been evaluated previously in numerous RCTs^47,48^ and we here sought to maximize the number of subjects receiving CBT in order to reveal treatment effects on cellular markers with sufficient statistical power. Nevertheless, we included multiple baseline assessments to control for standard confounds related to time and repeated testing, noting no changes between the two baselines, marked symptom improvement after treatment onset and a significant association between compliance to exposure exercises and anxiety relief. A minority of the patients had concurrent SSRI medication, but results remained significant after adding this as a nuisance variable. Thus, it is reasonable to assume that improved symptomatology is explained mainly by the psychological intervention alone. Considering the stable pharmacotherapy and low number of concurrent mental and somatic illnesses, other pharmacological explanations to the current effects on telomerase activity and GPx are also unlikely. Telomerase activity was measured in peripheral blood mononuclear cells, therefore, possible changes in telomerase, but not GPx, could be confounded by changes in the cellular subtype composition in the blood samples obtained. However, the ratio of these cell types was added as nuisance variable, and the results did not alter accordingly.

Apart from further validating internet-delivered CBT as an effective psychological treatment for SAD, our findings suggest improvement in indices of cellular health in tandem with mental health. The current findings are novel and need to be replicated, but provide initial evidence for the notion that biological processes related to cellular protection are involved in the response to a psychosocial intervention for anxiety and are also potentially useful for treatment prediction. Identifying biological correlates of successful treatments, at the cellular level, could advance our understanding of the mechanisms underlying remission, and pave the way for the development of novel treatment options.

## Supplementary information

Supplementary Material

## Data Availability

All the STATA statistical software commands are available upon request to the corresponding author.
