# Supplementary material for: Improvement in indices of cellular protection after psychological treatment for social anxiety disorder
Source: Transl Psychiatry. 2019 Dec 19;9:340. doi: 10.1038/s41398-019-0668-2 (PMC6920472; doi:10.1038/s41398-019-0668-2)
Supplement: Supplementary file 1 — Supplementary Material [file 41398_2019_668_MOESM1_ESM.docx]

# Supplementary Information

# Improvement in indices of cellular protection after psychological treatment for social anxiety disorder

Kristoffer N.T. Månsson^a,b,c^, PhD*

Daniel Lindqvist^d^, MD, PhD

Liu L. Yang^e,f^, MD

Cecilia Svanborg^g^, MD, PhD

Josef Isung^g^, MD, PhD

Gustav Nilsonne^h,i^, MD, PhD

Lise Bergman-Nordgren^g^, PhD

Samir El Alaoui^g^, PhD

Erik Hedman-Lagerlöf^h^, PhD

Martin Kraepelien^g^, PhD

Jens Högström^g^, PhD

Gerhard Andersson^j,g^, PhD

Carl-Johan Boraxbekk^k,l^, PhD

Håkan Fischer^b^, PhD

Catharina Lavebratt^e,f^, PhD

Owen M. Wolkowitz^m^, MD

Tomas Furmark^c^, PhD

^a^ Centre for Psychiatry Research, Department of Clinical Neuroscience, Karolinska Institutet, Stockholm, Sweden

^b^ Department of Psychology, Stockholm University, Stockholm, Sweden

^c^ Department of Psychology, Uppsala University, Uppsala, Sweden

^d^ Department of Clinical Sciences Lund, Psychiatry, Lund University, Lund, Sweden

^e^ Department of Molecular Medicine and Surgery, Karolinska Institutet, Stockholm, Sweden

^f^ Center for Molecular Medicine, Karolinska University Hospital, Stockholm, Sweden

^g^ Centre for Psychiatry Research, Department of Clinical Neuroscience, Karolinska Institutet, & Stockholm Health Care Services, Region Stockholm, Stockholm

^h^ Department of Clinical Neuroscience, Karolinska Institutet, Stockholm, Sweden

^i^ Stress Research Institute, Stockholm University, Stockholm, Sweden

^j^ Department of Behavioural Sciences and Learning, Linköping University, Linköping, Sweden

^k^ Centre for Demographic and Ageing Research, Umeå University, Umeå, Sweden;

^l^ Center for Magnetic Resonance (DRCMR), Centre for Functional and Diagnostic Imaging and Research, Copenhagen University Hospital, Hvidovre, Denmark

^m^ Department of Psychiatry, University of California, San Francisco, San Francisco, United States

***Address for correspondence:** Kristoffer N T Månsson, Centre for Psychiatry Research, Department of Clinical Neuroscience, Karolinska Institutet, Stockholm, Sweden. Tel: +46 (0) 70 580 3267; e-mail: kristoffer.mansson@ki.se

## METHODS AND MATERIALS

### Assays

Experienced research nurses collected all blood samples in the morning (from 7:00 am to 11:30 am) after patients had been fasting since 10:00 pm the night before. To ensure compliance, all the patients received a mobile phone text message the day before to remind about fasting instructions. The patients were resting for 15 minutes before the blood was obtained.

Whole blood was obtained in 8 ml BD Vacutainer® CPT™ Mononuclear Cell Preparation Tube - Sodium Citrate (Becton Dickinson). Plasma and mononuclear cells, being lymphocytes and monocytes, were separated within 15 min to 2 hours of sampling according to manufacturer’s protocol. Briefly, whole blood was centrifuged for 20 min at 1500 g, whereafter plasma and the mononuclear cell layer were separated and centrifuged again for 15 min at 300 g with PBS added to the cells for washing. The plasma was immediately frozen at -80°C. Approximately half of the pelleted cells were lysed by incubation with 120µl CHAPS (Merck Millipore, including 0.15 units/µl RiboLock [LifeTechnologies, Thermo Fisher Scientific]) on wet ice for 30 min and three short vortexes, thereafter the lysate was stored at -80°C.

Approximately half of the pelleted cells were stored at -80°C until DNA extraction. Genomic DNA was extracted using DNeasy® Blood & Tissue Kit (Qiagene), with a modified protocol to reduce DNA shearing. Briefly, cell lysis was done at 37°C for 3h, vortex was avoided, and centrifugations were performed at 6000g.[^1^](https://paperpile.com/c/XQXXPe/zbGUI) DNA concentration was quantified with NanoDrop ND-1000 Spectrophotometer (Nano-Drop Technologies Inc., Wilmington, DE, USA).

Differential leukocyte counts were determined in whole blood collected in ethylenediaminetetraacetic acid (EDTA) tubes by the University Hospital of Umeå.

#### Telomerase activity assay

Telomerase activity was assayed by modified real-time telomeric repeat amplification protocol (RT-TRAP).[^2^](https://paperpile.com/c/XQXXPe/4YUYb) Thawed lysate was centrifuged at 4°C at 12000 g for 20 min and the supernatant was transferred to an empty tube. Total protein concentration was measured in the supernatant by the DC Protein Assay (Bio-Rad) and all patients’ cell lysates were diluted to equal concentrations in CHAPS, aliquoted and refrozen at -80°C. An aliquot per sample was thawed and equal amounts of total protein (3.68 µg) from each sample was added to a reaction mix with a total volume of 50 µL containing 1 mM of each dNTP, 20 mM Tris-HCl (pH 8.3), 2.5 mM MgCl2, 63 mM KCl, 0.05% Tween 20, 1 mM EGTA, 8 µmol of each of the primers TS (5’-AATCCGTCGAGCAGAGTT-3’) and ACX (5’- GCGCGG(CTTACC)3CTAACC-3’). HepG2 (hepatic cancer) and ARO (thyroid cancer) cell lines were used as telomerase-positive controls, whereas CHAPS buffer and heat-inactivated samples were used as negative controls. TSR8 is an oligonucleotide with a sequence identical to the TS primer extended with 8 telomeric repeats being AG(GGTTAG)_7_. Serial dilutions of TSR8 control template were used to generate a standard curve to calculate telomerase activity. The serial dilutions were 0.2 amoles/μL, 0.04 amoles/μL, 0.008 amoles/μL, 0.0016 amoles/μL, 0.00032 amoles/μL, and 0.000064 amoles/μL corresponding to 200, 40, 8, 1.6, 0.32 and 0.064 TPG units/μL; TPG is the total product generated, corresponding to the number of TS primers (1 unit equals 10^-3^ amoles or 600 molecules) that are extended with at least 3 TTAGGG repeats by telomerase in the extract in a 30 min incubation at 30°C. The reaction mix was incubated at 30°C for 30 min followed by termination at 95°C for 5 min. Then 8 µL of the telomeric repeat products were used for the RT-TRAP assay amplified by 8 µL Power SYBR Green in 384-well plates. The reaction was performed on QuantStudio 7 Flex (Applied Biosystems; Life Technologies; Thermo Fisher Scientific Inc.) with the following conditions: 95°C for 10 min, followed by 35 repeats of 95°C for 20 s, 52°C for 30 s and 72°C for 60 s. Samples, controls and standard curve dilutions were run in triplicate, standard curve and controls on all plates. All samples from each patient were run on the same plate. The telomerase activity is given as [units/µl] in 0.588 µg of total protein ([3.68*8]/50). Efficiency was 95-101%. The correlation coefficients of the standard curves were above 0.98. The coefficients of variation (CV) of intra-assay Ct values for the standard dilutions of the four plates was = 1.0% and inter-assay was = 0.47%. Detection success rate was 100%, all samples run in the same batch.

Three samples were not included in the telomerase activity analysis because two of them had too low protein concentration from the cell lysate, and one had no protein in the cells. These samples were all obtained at the first baseline, and thus, the second baseline was used as the pretreatment assessment point for these patients.

#### Glutathione peroxidase activity assay

Glutathione peroxidase (GPx) activity was determined using BioVision Glutathione Peroxidase Activity Colorimetric Assay Kit (Catalog#K726-100) according to the protocol, where the GPx activity was calculated using an NADPH standard curve. In brief, each plasma sample (15 μl) diluted with assay buffer to 50 μl was in duplicates assessed for GPx activity, with all samples per patient run within the same 96-well plate. Forty microliter reaction mix (33 μl Assay buffer, 3 μl 40 mM NADPH, 2 μl Glutathione reductase solution and 2 μl Glutathione (GSH) solution) was added to each well and incubated at 25°C for 15 min to deplete all oxidized glutathione (GSSG) in the sample. At this point, all samples had an optical density (OD) value >1 at 340 nm. Then 10 μl Cumene hydroperoxide solution was added to each well and reaction kinetics at 25°C were followed for 25 min at 340 nm. A standard curve of NADPH (from 0 nmol/well to 60 nmol/well) was run in duplicate on each plate to enable determine the GPx activity for each sample. Pooled GPx positive control from 6 assay kits kept in -20°C without repeated freeze-thawing and negative control (NC) were run in duplicate on each plate, which allowed controlling for variation between and within plates. The kinetics curve for each sample was checked to assure linearity and maximum time interval between T1 and T2 (all but a few samples had T1=5 min and T2=25 min). The NADPH amount change B between T1 and T2 was determined by applying [(OD_T1_- OD_T2_)-(NC_ OD_T1_-RC_OD_T2_)] to NAPDH standard curve for each sample. The GPx activity was calculated as B/[(T2-T1)*sample volume]=nmol/min*ml. The correlation coefficients of the standard curves were all above 0.99. The inter-plate CV of GPx activity was 6.3% and the within-plate CV was 4.4% calculated from positive control run in nine 96-well plates. The assay success rate was 100% (*n*=46 at each time-point), and all samples were run in the same batch.

#### Relative telomere length assay

Relative telomere length was determined using real-time quantitative PCR (qPCR) according to Cawthon et al’s protocol[^3^](https://paperpile.com/c/XQXXPe/Ywo3x) where the relative telomere to single copy gene (T/S) ratios was determined using a standard curve. In brief, each DNA sample (10 ng) was assessed for the telomere and the single-copy gene (hemoglobin-b, *HBB* ) in triplicate within the same 384-well plate, amplified by using Platinum® SYBR® Green and 0.5 µM of each primer in 10 µl total reaction volume. The reaction was performed on QuantStudio 7 Flex (Applied Biosystems; Life Technologies, Carlsbad, CA, USA) with the following conditions: 50°C for 2 min, then 95°C for 10 min, followed by 40 repeats of 95°C for 15 s and 60°C for 1 min, followed by a dissociation stage to monitor amplification specificity. The same standard curve of pooled DNA from these patient samples ranging from 80 ng to 0.128 ng, was run on each plate for both amplicons and was used to determine the quantity of each amplicon for each sample. This allowed controlling for differences in the efficiencies between that of *Tel* and *HBB*. The amplicon quantities were then used to determine the T/S ratio for each sample. DNA samples with a Ct standard deviation of ≥ 0.35 between triplicates or a Ct value outside the standard curve were omitted from the analyses. The correlation coefficients of the standard curves were above 0.99 for each primer set and 384-plate. The inter-plate CV of T/S ratio was 6.3% calculated from a patient sample run in four 384-well plates. The telomere analysis detection success rate was 100%. The primer sequences were (written 5’ to 3’): Tel1: CGGTTTGTTTGGGTTTGGGTTTGGGTTTGGGTTTGGGTT; Tel2: GGCTTGCCTTACCCTTACCCTTACCCTTACCCTTACCCT; *HBB* Fw: GCTTCTGACACAACTGTGTTCACTAGC; *HBB* Rv: CACCAACTTCATCCACGTTCACC.

Samples from all 3 timepoints per individual were assayed in the same 384-well plate.

One sample was excluded from statistical analyses because of an issue related to labeling the tube when the blood was obtained. This sample was obtained at the first baseline, and therefore this patients second sample (second baseline) was used as the pretreatment assessment point.

## Discussion

In search for potential mechanisms by which increases in telomerase and GPx may contribute to superior treatment response, we note that GPx[^4^](https://paperpile.com/c/XQXXPe/QSaXP) and telomerase[^5^](https://paperpile.com/c/XQXXPe/AZnfs) activities, and telomere length[^6–8^](https://paperpile.com/c/XQXXPe/GuLIW+m9Mxg+2YKbr) could be related to brain morphology, brain development, response to injury and cellular changes in brain tissue. One study, for instance, demonstrated decreased levels of GPx in prefrontal cortex cells in post-mortem brains of depressed patients [^9^](https://paperpile.com/c/XQXXPe/kLn3z). Connections between the medial prefrontal cortex and the amygdala are parts of the neurocircuitry of emotion regulation[^10^](https://paperpile.com/c/XQXXPe/rRORT) and this link has shown to be disrupted in anxiety disorders.[^11^](https://paperpile.com/c/XQXXPe/NOBId) In previous studies we found anxiety-related changes in amygdala neural responsivity and brain morphology after CBT for SAD.[^12,13^](https://paperpile.com/c/XQXXPe/Edq2j+zLP3Z) It could be speculated that CBT and indices of cellular aging are linked to concomitant alterations in anxiety-related brain parameters, although this would need to be tested in future research.

## References

1 [O’Callaghan NJ, Fenech M. A quantitative PCR method for measuring absolute telomere length. *Biol Proced Online* 2011; **13**: 3.](http://paperpile.com/b/XQXXPe/zbGUI)

2 [Hou M, Xu D, Björkholm M, Gruber A. Real-time quantitative telomeric repeat amplification protocol assay for the detection of telomerase activity. *Clin Chem* 2001; **47**: 519–524.](http://paperpile.com/b/XQXXPe/4YUYb)

3 [Cawthon RM. Telomere measurement by quantitative PCR. *Nucleic Acids Res* 2002; **30**: e47.](http://paperpile.com/b/XQXXPe/Ywo3x)

4 [Cardoso BR, Hare DJ, Bush AI, Roberts BR. Glutathione peroxidase 4: a new player in neurodegeneration? *Mol Psychiatry* 2017; **22**: 328–335.](http://paperpile.com/b/XQXXPe/QSaXP)

5 [Grin Y, Admoni T, Priel E. Telomerase activity in the various regions of mouse brain: non-radioactive telomerase repeat amplification protocol (TRAP) assay. *J Vis Exp* 2014; : e51865.](http://paperpile.com/b/XQXXPe/AZnfs)

6 [Nilsonne G, Tamm S, Månsson K, Åkerstedt T, Lekander M. Leukocyte telomere length and hippocampus volume: a meta-analysis. *F1000Res* 2015; **4**: 1073.](http://paperpile.com/b/XQXXPe/GuLIW)

7 [King KS *et al.* Effect of leukocyte telomere length on total and regional brain volumes in a large population-based cohort. *JAMA Neurol* 2014; **71**: 1247–1254.](http://paperpile.com/b/XQXXPe/m9Mxg)

8 [Jacobs EG, Epel ES, Lin J, Blackburn EH, Rasgon NL. Relationship between leukocyte telomere length, telomerase activity, and hippocampal volume in early aging. *JAMA Neurol* 2014; **71**: 921–923.](http://paperpile.com/b/XQXXPe/2YKbr)

9 [Gawryluk JW, Wang J-F, Andreazza AC, Shao L, Young LT. Decreased levels of glutathione, the major brain antioxidant, in post-mortem prefrontal cortex from patients with psychiatric disorders. *Int J Neuropsychopharmacol* 2011; **14**: 123–130.](http://paperpile.com/b/XQXXPe/kLn3z)

10 [Hartley CA, Phelps EA. Changing fear: The neurocircuitry of emotion regulation. *Neuropsychopharmacology* 2009; **35**: 136–146.](http://paperpile.com/b/XQXXPe/rRORT)

11 [Rauch SL, Shin LM, Phelps EA. Neurocircuitry Models of Posttraumatic Stress Disorder and Extinction: Human Neuroimaging Research—Past, Present, and Future. *Biol Psychiatry* 2006; **60**: 376–382.](http://paperpile.com/b/XQXXPe/NOBId)

12 [Månsson KNT](http://paperpile.com/b/XQXXPe/Edq2j) [*et al.* Neuroplasticity in response to cognitive behavior therapy for social anxiety disorder. *Transl Psychiatry* 2016; **6**: e727.](http://paperpile.com/b/XQXXPe/Edq2j)

13 [Furmark T](http://paperpile.com/b/XQXXPe/zLP3Z) [*et al.* Common changes in cerebral blood flow in patients with social phobia treated with citalopram or cognitive-behavioral therapy. *Arch Gen Psychiatry* 2002; **59**: 425–433.](http://paperpile.com/b/XQXXPe/zLP3Z)
